# Mechanisms of action of the BCL-2 inhibitor venetoclax in multiple myeloma: a literature review

**DOI:** 10.3389/fphar.2023.1291920

**Published:** 2023-11-06

**Authors:** Qiang Cao, Xinyan Wu, Qi Zhang, Junling Gong, Yuquan Chen, Yanwei You, Jun Shen, Yi Qiang, Guangzhu Cao

**Affiliations:** ^1^ Department of Earth Sciences, Kunming University of Science and Technology, Kunming, China; ^2^ Department of Pharmacy, Nanjing Drum Tower Hospital, Affiliated Hospital of Medical School, Nanjing University, Nanjing, China; ^3^ College of Veterinary Medicine, Sichuan Agricultural University, Chengdu, China; ^4^ Undergraduate Department, Taishan University, Taian, China; ^5^ School of Public Health, Nanchang University, Nanchang, China; ^6^ Institute of Medical Information/Library, Chinese Academy of Medical Sciences, Beijing, China; ^7^ Division of Sports Science & Physical Education, Tsinghua University, Beijing, China

**Keywords:** cell apoptosis, multiple myeloma, anti-cancer drug, cell cycle, targeted therapies

## Abstract

Abnormal cellular apoptosis plays a pivotal role in the pathogenesis of Multiple Myeloma (MM). Over the years, BCL-2, a crucial anti-apoptotic protein, has garnered significant attention in MM therapeutic research. Venetoclax (VTC), a small-molecule targeted agent, effectively inhibits BCL-2, promoting the programmed death of cancerous cells. While VTC has been employed to treat various hematological malignancies, its particular efficacy in MM has showcased its potential for broader clinical applications. In this review, we delve into the intricacies of how VTC modulates apoptosis in MM cells by targeting BCL-2 and the overarching influence of the BCL-2 protein family in MM apoptosis regulation. Our findings highlight the nuanced interplay between VTC, BCL-2, and MM, offering insights that may pave the way for optimizing therapeutic strategies. Through this comprehensive analysis, we aim to lay a solid groundwork for future explorations into VTC’s clinical applications and the profound effects of BCL-2 on cellular apoptosis.

## 1 Introduction

Multiple Myeloma (MM) is a clonal plasma cell malignancy characterized by the malignant proliferation of monoclonal terminally differentiated plasma cells in the bone marrow or extramedullary sites (García-Ortiz et al., 2021; Neumeister et al., 2022; Fend et al., 2023). MM is typified by the secretion of monoclonal immunoglobulins by abnormal plasma cells, leading to end-organ damage and presenting characteristic clinical manifestations such as hypercalcemia, renal failure, anemia, and osteolytic bone destruction (Gupta et al., 2020; Heider et al., 2021; Urban et al., 2023). B-cell lymphoma-2 (BCL-2), a key anti-apoptotic protein in intrinsic programmed cell death, is often overexpressed in malignant hematological diseases, including MM. Its overexpression is associated with tumor progression and increased resistance to conventional chemotherapy and immunotherapies, thereby becoming a major focus of hematological cancer research in recent years (Suvarna et al., 2019; Ramesh and Medema, 2020; Michalski et al., 2023).

Venetoclax (VTC) is a selective BCL-2 inhibitor that directly targets the BCL-2 protein, modulating the mitochondrial apoptotic pathway and inducing tumor cell death (Li et al., 2019; Reddy et al., 2021). It has demonstrated efficacy in various hematological malignancies. VTC is an orally administered drug with a comparatively safe route of administration. Compared to other BCL-2 inhibitors (such as Navitoclax), VTC has higher selectivity towards BCL-2, reducing the risk of thrombocytopenia and yielding better therapeutic outcomes with milder adverse effects. In patients with mild to moderate hepatic and renal dysfunction, the clearance rate of VTC is scarcely affected, indicating good tolerability in clinical applications (Roca-Portoles et al., 2020; Wang et al., 2020; Garg et al., 2021; Cao et al., 2023a). Lasica et al.‘s study (Lasica and Anderson, 2021) demonstrated that MM patients treated with VTC have a higher 5-year survival rate compared to those treated with conventional chemotherapy. Hashim et al.‘s study (Hashim et al., 2020) shown that the overall response rate in MM patients treated with VTC is as high as 84%. Multiple studies have collectively indicated that VTC can extend the lifespan of MM patients by regulating apoptosis in MM cells through the modulation of the BCL-2 protein family (Fairbrother et al., 2019; Basu, 2022; Diepstraten et al., 2022).

In this review, we delineate the mechanisms by which Venetoclax (VTC) modulates apoptosis in Multiple Myeloma (MM) cells, emphasizing its interaction with the BCL-2 protein. We also explore the significance of the BCL-2 protein family in regulating apoptosis within MM cells (Kapoor et al., 2020; Qian et al., 2022; Czabotar and Garcia-Saez, 2023). Our objective is to lay a foundation for further understanding of VTC’s clinical potential and the broader role of BCL-2 in cellular apoptosis.

## 2 BCL-2 protein family

BCL-2 was the first identified anti-apoptotic gene that prolongs cellular survival rather than promoting cellular proliferation. The BCL-2 protein family encompasses over 12 proteins and is primarily divided into three functional groups:

(1) Pro-Growth, Anti-Apoptotic Subfamily: Comprising proteins like BCL-2, BCL-XL, and BCL-W, this subfamily plays a critical role in promoting cell survival by inhibiting pro-apoptotic proteins. For instance, BCL-XL can sequester BAX, a pro-apoptotic protein, while BCL-2 primarily restricts BAX’s activity (Lopez et al., 2022; Rosa et al., 2022). In MM, overexpression of these proteins can make cancerous cells resistant to apoptosis, thereby contributing to disease progression.
(2) Pro-Apoptotic Subfamily with Multiple BH Domains: This group includes BAX, BAK, and BOK, which are central to initiating the intrinsic apoptosis pathway. BAK can associate with BCL-XL, whereas BAX predominantly binds with BCL-2 (Shalaby et al., 2020; Green, 2022). In MM, aberrations in the function or expression of these proteins can disrupt the balance between cell survival and death, enabling MM cells to evade apoptosis.
(3) Pro-Apoptotic Subfamily with Only a BH3 Domain: Encompassing proteins such as BIM, BIK, BAD, and NOXA, this subfamily is crucial for sensing cellular stress and damage signals. Their primary function is to induce apoptosis by binding and inhibiting anti-apoptotic proteins, thereby releasing pro-apoptotic proteins like BAX or BAK to initiate cell death. BIM, for example, shows versatility in binding with all anti-apoptotic proteins. Conversely, BAD opposes BCL-2, BCL-XL, and BCL-W, while NOXA has specificity for MCL-1 (Kurschat et al., 2021; Kaloni et al., 2023; Sarkar et al., 2023). In MM, understanding the interactions and imbalances among these proteins can offer insights into potential therapeutic targets.

In humans, apoptotic pathways primarily consist of the intrinsic (mitochondrial) and extrinsic (death receptor) apoptotic pathways. The former is triggered by intracellular damage, leading to the activation of BAX and BAK (Al-Aamri et al., 2021; Chaudhry et al., 2022; Han et al., 2023). These proteins assemble into pore structures between the inner and outer mitochondrial membranes, inducing Mitochondrial Outer Membrane Permeabilization (MOMP), which subsequently releases cytochrome C into the cytoplasm, ultimately leading to cellular apoptosis (Cao et al., 2022a; Wang et al., 2022; Bernardi et al., 2023). The latter pathway operates through the formation of a death-inducing signaling complex by the binding of death ligands to the death receptor FAS, thereby activating BAX and BAK to initiate apoptosis (Bedoui et al., 2020; Lossi, 2022; Kalkavan et al., 2023).

VTC as a BCL-2 inhibitor, binds with high specificity to the BH3 domain of the BCL-2 protein. This binding displaces pro-apoptotic proteins like BIM, enabling them to activate BAX and BAK, thereby initiating apoptosis. By directly antagonizing BCL-2’s anti-apoptotic action, Venetoclax reinstates the natural cellular process of apoptosis, particularly in cells like Multiple Myeloma cells that have aberrant BCL-2 expression.

## 3 Role of BCL-2 in the progression and development of multiple myeloma

The most common subtypes of MM are IgA, IgM, and IgG. In malignant tumors, elevated levels of BCL-2 can be driven by various mechanisms, such as chromosomal translocations, gene amplification, and the downregulation or deletion of microRNAs involved in degrading BCL-2 RNA. Some myelomas may also evade apoptosis by uncontrolled transcription of BCL-2 protein, facilitated by gene deletions or the amplification of the miR-17–92 cluster. In approximately 87% of IgG cases, the BCL-2 gene is found to be translocated downstream of the gene encoding the immunoglobulin heavy chain, leading to its overexpression (Bazarbachi et al., 2021; MAATAOUI et al., 2021; Wang et al., 2021; Gkoliou et al., 2023). About 35% of patients with MM exhibit BCL-2 overexpression, which is associated with a poor prognosis. In 41% of IgM cases, overexpression of BCL-2 is detectable due to the amplification at the 18q21 locus, and this is correlated with poor prognosis in IgM patients (Deng et al., 2019; Zhang et al., 2021; Yehia et al., 2023).

## 4 VTC treatment for multiple myeloma

**FIGURE 1:**
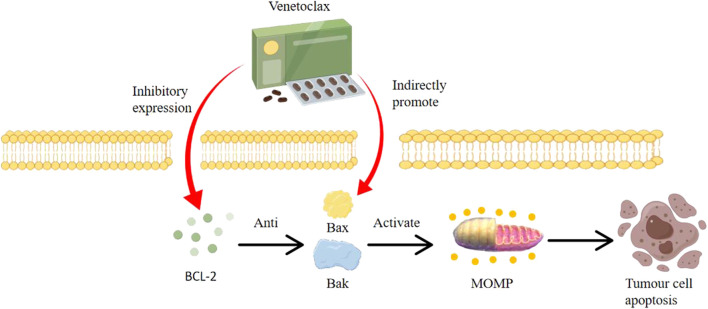
Mechanisms of cancer suppression by venetoclax (by Figdraw).

As shown in Figure 1, The BCL-2 inhibitor VTC is a highly selective oral BCL-2 inhibitor. Its primary mode of action involves activating BAK and BAX proteins, leading to mitochondrial outer membrane permeabilization (MOMP) and subsequent cell apoptosis (Kapoor et al., 2020; de Ridder et al., 2021; Rosa et al., 2022). By inhibiting cellular growth, VTC induces apoptosis in tumor cell lines, delays tumor progression, and extends overall survival. VTC is highly specific to BCL-2 and has low toxicity, displaying cytotoxicity across various hematologic malignancies, including MM. As the function of BCL-2 is non-essential in many normal cells and is commonly overexpressed in blood malignancies including MM, VTC has advantages over traditional cytotoxic drugs (Cao et al., 2022b; Soncini et al., 2022; Zhang et al., 2022).

### 4.1 Monotherapy with VTC

Recent investigations have highlighted the potential of Venetoclax (VTC) in the treatment of Multiple Myeloma (MM). A pivotal study by Bonolo et al. (Bonolo et al., 2020) revealed that VTC exhibits broad cytotoxic activity across various MM cell subtypes, emphasizing its versatile role in MM management. In a more direct clinical context, Ehsan et al. (Ehsan et al., 2021) undertook a study involving 135 MM patients to assess the therapeutic efficacy and tolerability of VTC when used as a standalone treatment. Within this cohort, 68 patients underwent a dose-escalation regimen (ranging from 300 to 1,000 mg/day), while the remaining 67 were administered a fixed dose of 1,000 mg/day. The outcomes were promising: Objective Response Rates (ORR) stood at 49%, and Complete Remission (CR) rates reached 12%. These results underscore a clinically recommended monotherapy dose of 1,000 mg/day for MM patients. Augmenting these findings, Cheikh et al. (Cheikh et al., 2023) proposed that VTC monotherapy might serve as a bridge to other robust therapeutic options for various MM presentations. Yet, it is essential to approach this suggestion with caution, considering potential limitations or biases inherent in single-study observations. These insights not only bolster the scientific understanding of VTC’s role but also hold significant implications for refining clinical strategies in MM patient care.

### 4.2 Combination therapy of VTC and chemotherapy

Numerous pre-clinical studies indicate that overexpression of BCL-2 is also associated with increased resistance to commonly used chemotherapeutic agents (Suvarna et al., 2019; Zhang et al., 2021; Alam et al., 2022; Qiang et al., 2022; Kaloni et al., 2023). *In vitro* studies show synergistic effects when VTC is combined with Rituximab, with superior efficacy compared to Rituximab alone (Juárez-Salcedo et al., 2019; Yue et al., 2020; Vogiatzi et al., 2022). A study by Samineni et al. (Samineni et al., 2022) has established the safety, efficacy, and potential of VTC combined with R-CHOP chemotherapy in treating MM, setting a paradigm for the administration strategy of VTC + R-CHOP. This will serve as a reference for the frontline treatment of MM with VTC (Zelenetz et al., 2019; Gomez et al., 2020; Yue et al., 2020).

### 4.3 Combination therapy of VTC with other targeted drugs in MM treatment

Yue et al. conducted a study (Yue et al., 2020) exploring the efficacy and tolerability of rituximab in combination with VTC in a mouse model of MM. The findings delineated that following a 4-week intervention period, there was a significant diminution in both the average neoplastic mass dimension and weight in the murine subjects administered with a combination of rituximab and VTC, when juxtaposed with the control group, as well as the groups receiving monotherapy of either agent. Additionally, the regimen was well tolerated by all participants in the experimental cohort. Subsequent to the therapeutic regimen incorporating rituximab in conjunction with VTC, there was a pronounced decrement in oncogenic cellular proliferation coupled with an augmentation in programmed cellular death, indicative of the prospective utility of this combined therapeutic approach in managing patients afflicted with MM (Dalton et al., 2021; Niemann et al., 2021; You et al., 2023). An investigation spearheaded by Salcedo et al. (Juárez-Salcedo et al., 2019) assessed the efficacy of amalgamating VTC and ibrutinib in the therapeutic management of MM, delineating a marked superiority over singular therapeutic strategies. The emerging data advocates for the prospective role of this combination as an intermediary therapeutic strategy preceding transplantation, albeit necessitating validation through expansive clinical trials. Kocoglu et al.‘s study (Kocoglu and Badros, 2020) ascertained that the ORR and Complete Remission (CR) rate in the therapeutic management of MM utilizing an amalgamation of VTC, rituximab, and bortezomib, notably surpassed the outcomes garnered from the utilization of VTC as a monotherapeutic strategy. In an intriguing juxtaposition, while the IgG subtype demonstrates heightened activity, the more quiescent IgA subtype manifested superior ORR and CR metrics. This infers that the combined therapeutic regimen of VTC, rituximab, and bortezomib not only exhibits an enhanced efficacy and safety profile but also delineates differential responsiveness across varied MM subtypes. Such findings furnish invaluable insights, laying the groundwork for subsequent scholarly endeavors to delve deeper into combinatory therapeutic strategies encompassing VTC and other precision-targeted pharmaceutical agents (Nuvolone et al., 2021; Al-Mansour, 2022; Wu et al., 2023).

Current the study on MM treatment involving combinations of VTC with other targeted therapies is ongoing. Chu et al.‘s study (Chu et al., 2021) evaluated the safety and effectiveness of a triple regimen of VTC, lenalidomide, and rituximab for MM treatment and preliminarily confirmed its favorable therapeutic outcome. Some studies reported that the ORR was 78.5% for refractory relapsed inert MM patients when VTC was combined with atezolizumab and ocrelizumab, and the remission was sustained. They suggests that the combination of VTC with various targeted drugs holds substantial potential for MM treatment. However, given the relatively recent market introduction of VTC, ongoing safety evaluations are necessary to assess the safety of combining VTC with various drugs (Batta et al., 2020; Carneiro and El-Deiry, 2020; Nguyen et al., 2022; Cao et al., 2023b).

## 5 Prevention and management of VTC resistance and adverse reactions

VTC has shown significant promise in treating hematological malignancies, but a challenge has arisen in the form of secondary resistance observed in some MM patients after prolonged VTC treatment. The molecular mechanisms underpinning this resistance are warrant a deeper exploration. Several hypotheses have been postulated to explain VTC resistance. At the forefront of these is the emergence of missense mutations either in BCL-2 or BAX. Mutations in BCL-2 disrupt the binding of the BH3 domain with VTC. This interruption impedes the drug’s ability to trigger apoptosis, resulting in drug resistance. In contrast, mutations in BAX affect its anchoring to the mitochondrial membrane. Such mutations not only confer resistance to VTC but also lead to cross-resistance to other anti-tumor agents (Wei et al., 2020; Lasica and Anderson, 2021; Cao et al., 2023c).

Recent study offers further insights. A study by Prado et al. (Prado et al., 2021) highlighted a mechanism involving the sequestration of the pro-apoptotic protein, BIM. This process is initiated after the upregulation of BCL-XL expression in VTC-resistant MM cell lines. It suggests that an increased expression of certain anti-apoptotic proteins could be pivotal in both acquired and intrinsic VTC resistance (Gupta et al., 2021; Liu et al., 2022; Cao et al., 2023b; Granau et al., 2023). In light of these findings, Satta et al.‘s comparative study (Satta and Grant, 2020) becomes crucial. Their analysis underscored the potential of combination therapies in circumventing VTC resistance. Particularly, they posited that a regimen combining VTC with rituximab might offer enhanced efficacy compared to other therapeutic combinations (Oriol et al., 2020; Chu et al., 2021).

VTC treatment, whether as a monotherapy or in combination with other therapeutic agents, is associated with several common adverse reactions. These include nausea, diarrhea, neutropenia, fatigue, and notably, thrombocytopenia. Among these, hematological toxicities, such as neutropenia and thrombocytopenia, are more commonly observed than non-hematological side effects. Notably, the incidence of hematological toxicities does not markedly increase when VTC is used in conjunction with other treatments. Despite VTC’s pronounced affinity for BCL-2 over BCL-XL and BCL-W, thrombocytopenia remains a prevalent side effect during treatment (Delou et al., 2019; Sarkozy and Ribrag, 2020; Van Wagoner et al., 2023). To manage this, regular platelet count monitoring is recommended, with dosage adjustments or temporary discontinuation in severe cases. Growth factors or platelet transfusions might also be employed as supportive measures.

Another critical adverse effect to consider is the risk of tumor lysis syndrome (TLS) with VTC. TLS is predominantly linked to the patient’s tumor burden and renal function. Its onset is typically heralded by a swift reduction in tumor cell counts. To mitigate TLS, clinicians must tailor VTC’s administration based on tumor load. Measures include the early introduction of anti-hyperuricemic agents, such as allopurinol or rasburicase, and ensuring aggressive hydration to prevent renal complications (Tambaro and Wierda, 2020; Wierda et al., 2021; Arora et al., 2022).

## 6 Conclusion and future outlook

VTC offers an innovative treatment approach for patients with relapsed and refractory MM, showing good tolerability across different MM subtypes. The varying patterns and levels of BCL-2 expression may account for the heterogeneous response to VTC among different MM subtypes. While the efficacy of VTC as a monotherapy in MM treatment remains limited, combination therapy with other treatment modalities has proven to yield better outcomes for MM patients. Current research suggests that VTC can serve as a bridging treatment prior to other more beneficial therapies (such as hematopoietic stem cell transplantation) for MM patients. Clinical studies are continually emerging that demonstrate the immense potential of VTC in combination with other targeted therapies for MM treatment. Resistance to VTC may be associated with mutations in BCL-2 family genes or increased expression of anti-apoptotic proteins. Combining VTC with other targeted therapies may mitigate this resistance to a certain extent.

The development, progression, and drug resistance of MM are closely related to the overexpression of BCL-2 family proteins. The BCL-2 inhibitor, VTC, provides a novel avenue for MM treatment, especially given its higher efficacy and safety when used in combination with other targeted therapies. However, due to its relatively recent market introduction and the limited amount of multicenter clinical data available, further prospective multicenter research is needed to delve deeper into the safety, efficacy, and additional combination strategies of VTC.
